# Jottings

**Published:** 1909-11-06

**Authors:** 


### JOTTINGS.

Mr. F. C. Raphael has been appointed Consulting Engineer to St. Bartholomew's Hospital.

The tender of Mr. C. E. Skinner, of Chatham, for the new Maidstone Infectious Hospital has been accepted at £3,540.

The Camberwell Guardians have laid out a lawn tennis court in the infirmary grounds for the use of the convalescent pauper inmates.

The King formally opened the new Jubilee Extension of the National Hospital for the Paralysed and Epileptic in Queen Square, Bloomsbury, on Thursday last.

We understand that the drainage of the Swanley Convalescent Home is being reconstructed and the home will be closed for patients during the progress of the work.

The Worshipful Company of Skinners has contributed £1,000 to the capital fund of St. Bartholomew's Hospital.

Sir Edgar Speyer, Bart., will take the chair at the Festival Banquet of the Great Northern Central Hospital, Holloway, N., which is to be held at the Whitehall Rooms, Hôtel Métropole, on Wednesday evening, November 24.

A bazaar in aid of the Gateshead Children's Hospital was opened last week in the Town Hall, Gateshead, by Councillor W. J. Sanderson, Newcastle, and realised the sum of £450 11s. 4d.

Owing to ill-health, Mr. Charles Wightman has resigned the chairmanship of the Evelina Hospital for Children, a position which he has held for some years. Mr. Donald Malcolm Scott has been chosen as his successor.

A special general meeting of Governors of the Poplar Hospital for Accidents, Poplar, E., was held at the hospital on Tuesday, November 2, for the purpose of authorising the disposal of certain stocks, funds, securities, and property belonging to the hospital.

The Commissioners of Public Works have sanctioned a loan of £8,000 to the Cork Joint Hospital Board for the purpose of providing a sanatorium, the loan to be repaid in twenty-five years, with interest at the rate of 3½ per cent. per annum, and to be issued by the Joint Board in instalments, the first being £5,000.

A scheme for the provision of a new sanatorium for male consumptives has been approved by the Blackburn Board of Guardians. It was decided to convert an old building in the workhouse district, formerly used as a smallpox hospital, for this purpose, and seven or eight beds will be provided.

At the last quarterly meeting the following gentlemen were elected Life Governors of the Leicester Infirmary:—Messrs. James Eastwood Pickard and Albert Pickard, in respect of their contributions to the respective extension funds, making £1,000 in all; Mr. W. G. Waterhouse Reynolds, Birstall Holt, in respect of his donation of £105 towards the extension fund; Mr. Hugh Faulkner, London Road, Leicester, in respect of his donation of £100 to the extension fund; Mr. John Norman, Claremont House, Birstall, in respect of his donation of £52 10s. to commemorate his seventieth birthday on August 31 last.

